# Mass Spectometry–Based SARS Genotyping

**DOI:** 10.1371/journal.pmed.0020052

**Published:** 2005-02-22

To quickly control infectious disease outbreaks, extensive information is required to identify the source and transmission routes, and to evaluate the effect of containment policies. Traditionally, scientists have used travel- and contact-tracing methods, but the recent SARS epidemic showed that sequence-based techniques for pathogen detection can also be important tools to help understand outbreaks. Jianjun Liu and colleagues adapted mass spectrometry (MS)–based genotyping, already used as a high-throughput way of detecting single nucleotide polymorphisms in human DNA, to the analysis of the SARS virus from clinical samples.

The major breakthroughs against SARS were the discovery of the SARS coronavirus (SARS-CoV) as the etiological agent and the sequencing of the SARS genome. Liu's colleagues at the Genome Institute of Singapore had previously shown that common genetic variants in the SARS-CoV genome could be used as molecular fingerprints to help trace the route of infection. However, as “sequence analysis of large numbers of clinical samples is challenging, cumbersome, and expensive,” they felt that “what is needed is a rapid, sensitive, high throughput, and cost-effective screening method.” Towards this goal, Liu and colleagues now demonstrate that an MS-based technique can quickly yield accurate information on clinical isolates (in this case from the 2003 SARS outbreak in Singapore).[Fig pmed-0020052-g001]

**Figure pmed-0020052-g001:**
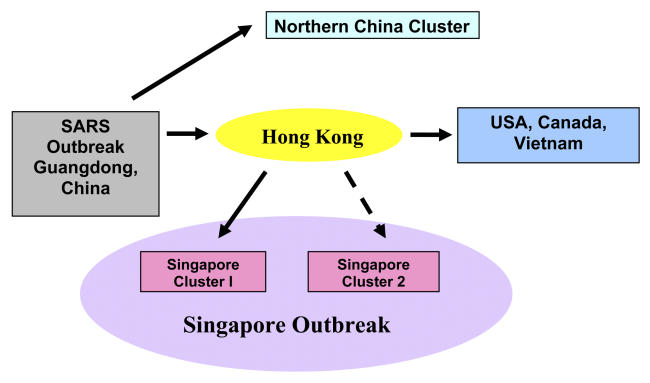
Two transmission routes for the Singapore SARS outbreak

The scientists demonstrate the sensitivity of the assay in detecting SARS-CoV variations and test it further in cultured viral isolates and uncultured lung tissue samples of SARS-CoV. They analyzed isolates taken from 13 patients with SARS at different stages of the Singapore outbreak, identified nine sequence variations, and discovered a new primary route of introduction of the virus into the Singapore population. They also found a Singaporean origin for a German case of SARS, a result that could not be derived from standard sequencing methods. The analysis of the uncultured lung tissue also found different sequences in a single patient, which suggested the presence of multiple viral sequence variants in one host.

The study suggests that MS-based genotyping can be used for large-scale genetic characterization of viral DNA from clinical samples. The researchers found that the method was accurate and sensitive, with a 95% success rat e for detecting sequence variations at low virus concentrations. The MS-based assay allows high-throughput analysis and complements the “gold standard” direct sequence analysis method, which is used to identify new sequence variations. As such, it is particularly useful for investigating agents for which extensive sequence information exists.

Liu and colleagues propose that the most efficient method for a large-scale population investigation would be initial characterization of a genome sequence by direct sequence analysis in a subset of samples, followed by MS-based analysis of informative genetic variations. Altogether, their results suggest that MS-based genetic analysis can help real-time investigations in disease outbreaks.

